# Lipopolysaccharide Lowers Cholesteryl Ester Transfer Protein by Activating F4/80^+^Clec4f^+^Vsig4^+^Ly6C^−^ Kupffer Cell Subsets

**DOI:** 10.1161/JAHA.117.008105

**Published:** 2018-03-10

**Authors:** Sam J. L. van der Tuin, Zhuang Li, Jimmy F. P. Berbée, Inge Verkouter, Linda E. Ringnalda, Annette E. Neele, Jan B. van Klinken, Sander S. Rensen, Jingyuan Fu, Menno P. J. de Winther, Albert K. Groen, Patrick C. N. Rensen, Ko Willems van Dijk, Yanan Wang

**Affiliations:** ^1^ Division of Endocrinology Department of Medicine Leiden University Medical Center Leiden The Netherlands; ^2^ Einthoven Laboratory for Experimental Vascular Medicine Leiden University Medical Center Leiden The Netherlands; ^3^ Department of Human Genetics Leiden University Medical Center Leiden The Netherlands; ^4^ Amsterdam Diabetes Center Department of Vascular Medicine Academic Medical Center University of Amsterdam The Netherlands; ^5^ Department of Pediatrics University of Groningen The Netherlands; ^6^ Department of Genetics University Medical Center Groningen University of Groningen The Netherlands; ^7^ Department of Medical Biochemistry Academic Medical Center Amsterdam The Netherlands; ^8^ Department of Surgery Maastricht University Medical Center Maastricht The Netherlands; ^9^ Institute for Cardiovascular Prevention (IPEK) Ludwig Maximilian's University Munich Germany

**Keywords:** inflammation, lipids and lipoprotein metabolism, liver, macrophage, marker, Inflammation, Lipids and Cholesterol, Biomarkers

## Abstract

**Background:**

Lipopolysaccharide (LPS) decreases hepatic CETP (cholesteryl ester transfer protein) expression albeit that the underlying mechanism is disputed. We recently showed that plasma CETP is mainly derived from Kupffer cells (KCs). In this study, we investigated the role of KC subsets in the mechanism by which LPS reduces CETP expression.

**Methods and Results:**

In CETP‐transgenic mice, LPS markedly decreased hepatic *CETP* expression and plasma CETP concentration without affecting hepatic macrophage number. This was paralleled by decreased expression of the resting KC markers C‐type lectin domain family 4, member f (*Clec4f*) and V‐set and immunoglobulin domain containing 4 (*Vsig4*), while expression of the infiltrating monocyte marker lymphocyte antigen 6 complex locus C (*Ly6C*) was increased. Simultaneously, the ratio of plasma high‐density lipoprotein‐cholesterol over non–high‐density lipoprotein‐cholesterol transiently increased. After ablation hepatic macrophages via injection with liposomal clodronate, the reappearance of hepatic gene and protein expression of CETP coincided with Clec4f and Vsig4, but not Ly6C. Double‐immunofluorescence staining showed that CETP co‐localized with Clec4f^+^ KCs and not Ly6C^+^ monocytes. In humans, microarray gene‐expression analysis of liver biopsies revealed that hepatic expression and plasma level of CETP both correlated with hepatic *VSIG4* expression. LPS administration decreased the plasma CETP concentration in humans. In vitro experiments showed that LPS reduced liver X receptor‐mediated CETP expression.

**Conclusions:**

Hepatic expression of CETP is exclusively confined to the resting KC subset (ie, F4/80^+^Clec4f^+^Vsig4^+^Ly6C^−^). LPS activated resting KCs, leading to reduction of Clec4f and Vsig4 expression and reduction of hepatic CETP expression, consequently decreasing plasma CETP and raising high‐density lipoprotein (HDL)‐cholesterol. This sequence of events is consistent with the anti‐inflammatory role of HDL in the response to LPS and may be relevant as a defense mechanism against bacterial infections.

**Clinical Perspective**

**What Is New?**

- Our findings show that hepatic CETP (cholesteryl ester transfer protein) is exclusively expressed by a resting Kupffer cell subset (ie, F4/80^+^Ly6C^−^Clec4f^+^) but not by activated macrophages or monocytes in the liver.
- Lipopolysaccharide exposure reduces hepatic CETP expression by activation of resting Kupffer cells, consequently decreasing plasma CETP and raising high‐density lipoprotein‐cholesterol.

**What Are the Clinical Implications?**

- This rapid conversion of the Kupffer cell subset by LPS, consequent loss of CETP expression and subsequent increase in plasma high‐density lipoproteins may play a role in the host defense against Gram‐negative bacterial infections.
- The strong association between the expression of CETP and activation markers by Kupffer cells implies that modulating high‐density lipoprotein metabolism via CETP inhibition may affect the inflammatory status of the liver.

## Introduction

Kupffer cells (KCs) are the resident tissue macrophages of the liver. KCs are characterized by specific surface proteins such as Clec4f (C‐type lectin domain family 4, member f) and Vsig4 (V‐set and immunoglobulin domain containing 4).^1^, ^2^, ^3^, ^4^ These surface markers distinguish KCs from other hepatic macrophages, which may have lost or have yet to acquire these KC markers. KCs play an important role in the response to various harmful agents such as intestine‐derived bacterial lipopolysaccharide (LPS) in the portal blood. LPS, a constituent of Gram‐negative bacteria, is a potent endotoxin that induces a strong cytokine‐mediated inflammatory response in the host.^5^ LPS activates KCs via the Toll‐like receptor 4 (TLR4) signaling pathway to release proinflammatory cytokines, such as tumor necrosis factor α (TNFα)^6^ and interleukin‐1β (IL‐1β).^7^ Therefore, KCs are crucial to detect LPS and trigger an antibacterial response.^8^, ^9^

We have recently shown that KCs are the main source of plasma cholesteryl ester transfer protein (CETP), and that plasma CETP concentration predicts hepatic macrophage content in humans.^10^ In that study, we also found that CETP was expressed by only a subset of hepatic macrophages in livers of both humans and mice transgenic for human CETP. Plasma CETP plays a pivotal role in the metabolism of high‐density lipoproteins (HDL) and (very‐) low‐density lipoproteins ((V)LDL) by mediating the exchange of cholesteryl ester for triglycerides (TG) between HDL and (V)LDL. Genetic deficiency for CETP increases plasma HDL‐cholesterol (C) and decreases cardiovascular events.^11^ This has led to the development of CETP inhibition as a potential strategy for the treatment of cardiovascular disease. Despite clearly favorable effects on the lipoprotein profile, pharmacological CETP inhibitors, such as torcetrapib,^12^ dalcetrapib^13^ and evacetrapib,^14^ failed to show clinical benefit on cardiovascular disease outcomes including atherosclerosis and vascular inflammation. While, recently, the Merck Company announced that the REVEAL (Evaluation of the Effects of Anacetrapib through Lipid Modification) study, which studies the effects of Anacetrapib on cardiovascular disease outcome met its primary end point, significantly reducing major coronary events defined as the composite of coronary death, myocardial infarction, and coronary revascularization.^15^ These results illustrate that the role and underlying mechanism of CETP in cardiovascular disease pathology is more complex than initially anticipated.

CETP not only has a role in lipid and lipoprotein metabolism, but also belongs to the family of lipid transfer/LPS‐binding proteins (LT/LBP).^16^ A previous study has demonstrated that CETP expression increases the mouse survival rate after injection of a lethal dose of LPS.^17^ In addition, LPS administration to CETP transgenic mice resulted in a rapid and marked decrease in plasma CETP concentration and hepatic CETP expression, accompanied by an increase in HDL‐C level.^18^ These data indicate that CETP may play a complex role in the response to LPS.

We previously observed that a subset of F4/80‐positive hepatic macrophages co‐express CETP.^10^ In the current study, we determined which subset of KCs is the predominant cellular source to the plasma CETP pool. Also, we investigated the role of KC subsets in the mechanism by which LPS reduces CETP expression. To this end, we determined the regulation of CETP in relation to hepatic macrophage markers after injection of LPS in APOE*3‐Leiden.CETP (E3L.CETP) mice and followed the kinetics of reappearance of CETP in relation to macrophage markers after removal of hepatic macrophages by liposomal clodronate. Furthermore, liver biopsies and plasma samples from 2 clinical studies were used to evaluate the CETP expression in KC subsets and effects of LPS on plasma CETP in humans.

## Methods

The data, analytic methods, and study materials will not be made available to other researchers for purposes of reproducing the results or replicating the procedure.

### Animals and Experimental Procedure

Female APOE*3‐Leiden.CETP (E3L.CETP) transgenic mice^19^ were housed under standard conditions with a 12‐hour light‐dark cycle and had free access to food and water during the experiment. At the age of 10 to 15 weeks, mice were fed a semi‐synthetic cholesterol‐rich diet, containing 15% (w/w) cacao butter, 1% (w/w) corn oil and 0.1% cholesterol (w/w, Western‐type diet; AB‐Diets, Woerden, The Netherlands) for a run‐in period of 6 weeks, followed by LPS injection or liposomal clodronate injection. Body weight and food intake were monitored during this study. The Institutional Ethics Committee for Animal Procedures from the Leiden University Medical Centre, Leiden, The Netherlands, approved the following studies.

### LPS Injection

After randomization according to plasma levels of triglycerides, total cholesterol, HDL‐C, body weight, and age, mice received an intraperitoneal injection of LPS (25‐μg per mouse; *Escherichia coli* serotype 055:B5, Sigma‐Aldrich) or vehicle (LPS‐free phosphate‐buffered saline, control group), and blood samples were drawn before and 8, 24, and 48 hours after the injection. In a second study, mice were terminated 4, 8, and 48 hours after the injection of LPS.

### Liposomal Clodronate Injection

After randomization according to plasma total cholesterol (TC), HDL‐C, triglycerides, body weight and age, ensuring that all mice were equally old when they were euthanized, mice received 2 intraperitoneal injections of liposomal clodronate (20 mg/kg bodyweight; purchased from Dr N. van Rooijen, Amsterdam) at a 3‐day interval to deplete macrophages from the liver.^10^ Blood samples were drawn before and after the second injection weekly up to 9 weeks. In a second study, mice were terminated 3 days (0 week), or 3, 6, or 9 weeks after the second injection. Control mice received no liposomal clodronate treatment.

### Blood Sampling, Plasma Lipid, and Lipoprotein Profiles

Blood was obtained via tail vein bleeding into heparin‐coated capillary tubes. The tubes were placed on ice and centrifuged, and obtained plasma was snap‐frozen in liquid nitrogen and stored at −80°C until further measurements. Plasma was assayed for triglycerides and cholesterol using the commercially available enzymatic kits 11488872 and 236691 (Roche Molecular Biochemicals, Indianapolis, IN, USA), respectively. To measure plasma HDL‐C, apoB‐containing lipoproteins were precipitated from plasma with 20% polyethylene glycol 6000 (Sigma Aldrich) in 200 mmol/L glycine buffer (pH 10) and HDL‐C was measured in the supernatant. Plasma non–HDL‐C was calculated by subtracting HDL‐C from plasma total cholesterol.

### Plasma CETP Concentration

Plasma CETP concentration was measured using the DAIICHI CETP ELISA kit according to manufacturer's instructions (Daiichi, Tokyo, Japan).

### Hepatic Gene Expression

Liver pieces were isolated and total RNA was extracted using the Nucleospin RNAII kit (Macherey‐Nagel) according to manufacturer's protocol. RNA concentration was determined by Nanodrop technology (Thermo Scientific). Total RNA was reverse‐transcribed with the iScript cDNA synthesis kit (Bio‐Rad) and qPCR was performed using a CFX96 (Bio‐Rad). Gene expression was normalized to Beta‐2 microglobulin (β*2m*), hypoxanthine ribosyltransferase (*Hprt*) and Beta‐actin (β*actin*). Relative expression was calculated and normalized to control group using Bio‐Rad CFX ManagerTM software 3.0 (Bio‐Rad). Primer sequences can be found in Table S1.

### Liver Histology

Paraffin‐embedded sections of mouse liver (5 μm) were stained for F4/80 and human CETP (ab51771; 1/1000, Abcam) as described previously,^10^ Clec4f (MAB2784; 1/1000, R&D Systems), Vsig4 (AF4674; 1/1000, R&D Systems) and Ly6C (ab15627; 1/400, Abcam). For immunofluorescence staining, the secondary antibodies donkey anti‐rabbit Alexa488 (A21206; Invitrogen) and goat anti‐rat Alexa555 (A21434; Invitrogen) were used. Finally, tissue sections were mounted with VECTASHIELD^®^ Mounting Medium with DAPI (Vector Laboratories). Positive cells were counted using a LeicaCTR5500 fluorescence microscope (Leica Microsystems GmbH). Representative pictures of immunostaining for CETP in liver sections of non‐CETP transgenic mice (APOE*3‐Leiden mice), APOE*3‐Leiden.CETP transgenic mice, and a healthy human donor are shown in Figure S1.

### Design of Human Studies

Ninety‐three severely obese subjects (BMI 30–74) underwent elective bariatric surgery from 2006 to 2009 at the Department of General Surgery, Maastricht University Medical Center, Maastricht, The Netherlands, as described.^20^ Subjects using anti‐inflammatory drugs or having acute or chronic inflammatory diseases, degenerative diseases, and subjects reporting alcoholic intake >10 g/day, were excluded. During surgery, liver biopsies were taken for mRNA isolation and in situ analyses. Venous blood samples were obtained after overnight fasting (≈8 hours) on the morning of surgery for analysis of the plasma CETP concentration. This study was approved by the Medical Ethics Board of Maastricht University Medical Centre, in line with the Declaration of Helsinki. All participants provided informed written consent.

The second study consisted of 20 healthy male subjects as described.^21^ Subjects with known genetic causes for low HDL‐cholesterol, secondary dyslipidemias such as familial combined hyperlipidemia, metabolic syndrome or secondary to hypertriglyceridemia, were excluded. On the morning of the study day at 7:30 am after an overnight fast, study participants were admitted to the research unit. At 7:45 am a catheter was inserted in an antecubital vein of each arm. At 8:00 am (time [t]=0), blood was drawn for baseline measurements. Subsequently, subjects received a bolus infusion of 1 ng/kg body weight of endotoxin (*E coli* LPS, catalog number 1235503, lot G2B274; United States Pharmacopeial Convention Inc, Rockville, MD) in the antecubital vein of the contralateral arm. The next morning at 8:00 am, 24 hours after endotoxin infusion, study participants returned after an overnight fast for the blood withdrawal. The study protocol was approved by the institutional review board at the Academic Medical Center in Amsterdam. Written informed consent was obtained from all subjects.

### In Vitro LPS Simulation in Human Monocyte‐Derived Macrophages

Peripheral blood mononuclear cells were isolated from a buffycoat (Sanquin blood supply, Amsterdam, the Netherlands) through density centrifugation using Lymphoprep™ (Axis‐Shield, Dundee, Scotland). Monocytes were then purified using human CD14 magnetic beads and MACS^®^ cell separation columns (Miltenyi Biotec, Bergisch Gladbach, Germany). Monocytes were plated in 24‐well tissue culture plates at a density of 1×10^6^ cells/mL (500 μL per well) and differentiated to macrophages for 6 days in Iscove's Modified Dulbecco's Medium (Sigma‐Aldrich) supplemented with 2 mmol/L l‐glutamine, penicillin (100 U/mL), streptomycin (100 μg/mL) and 10% fetal calf serum (All Gibco, Waltham, MA) in the presence of 50 ng/mL macrophage colony stimulating factor (MCSF) (Miltenyi Biotec, Bergisch Gladbach, Germany). On day 3, the medium was removed and substituted by fresh Iscove's Modified Dulbecco's Medium with 10% fetal calf serum and 50 ng/mL MCSF. On day 6, all medium was removed and replaced by Iscove's Modified Dulbecco's Medium with 10% fetal calf serum without MCSF and cells were activated for 18 hours with vehicle (DMSO), LPS (10 ng/mL, Sigma‐Aldrich), a liver X receptor (LXR) agonist (TO‐901317, 10 μmol/L, Sigma‐Aldrich), and LPS+LXR agonist. Total RNA was extracted from the cell lysate.

### Statistical Analysis

Data were analyzed by Graphpad Prism software, version 7, unless indicated otherwise. Significance of differences between the groups was calculated non‐parametrically using a Mann–Whitney *U* test for independent samples. All groups were compared with the control group. Bonferroni's method was used to determine significance in case of multiple comparisons. For the human LPS exposure study, the Student paired t test was used. To analyze the in vitro LPS exposure study, One‐way ANOVA was used, followed by the Fisher's Least Significant Difference (LSD) test to identify the differing groups. Spearman correlation was used to determine the correlations between parameters in both mouse and human studies. Linear regression analysis was performed on the associations between hepatic expression of CETP and macrophage markers and the coefficients of determination (*R* ^2^) were reported. For linear regression analysis of human liver microarray data, unadjusted Crude models (model 1) and a Model adjusted for age and sex (model 2) were applied using STATA Statistical Software, version 12.0. For experiments involving repeated measures of plasma lipids and CETP levels in the same animal, we used a linear mixed‐effects model with a heterogeneous first‐order autoregressive covariance matrix structure to model subject‐specific deviances from the group mean using IBM SPSS Statistical Software, version 23. Values are presented as means±SEM. *P* values <0.05 were considered statistically significant for single comparison.

## Results

### LPS Reduces Hepatic CETP Expression, Paralleled by Decreased Expression of Resting Kupffer Cell Markers and Increased Expression of Macrophage Activation Markers

**Figure 1:**
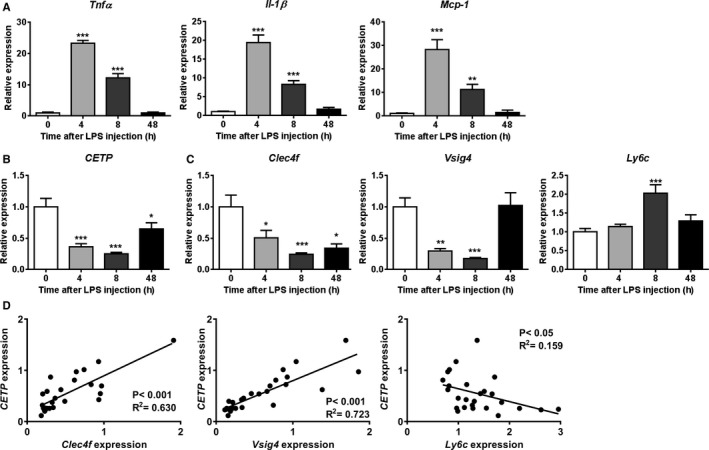
LPS reduces hepatic CETP expression, paralleled by decreased expression of resting Kupffer cell markers and increased expression of macrophage activation markers. Female APOE*3‐Leiden.CETP mice fed a Western‐type diet were intraperitoneally injected with 25 μg LPS, after which mice were euthanized at the indicated time points. Livers were assayed for mRNA of (A) *Tnfa*,* Il‐1*β, *Mcp‐1*; (B) *CETP* and (C) *Clec4f*,* Vsig4*,* Ly6C*. The correlations between hepatic mRNA expression of *CETP* with *Clec4f*,* Vsig4* and *Ly6C* were performed, and the goodness of fit *R* ^2^ from linear regression analyses were shown (D). For (A through C) data are presented as means±SEM (0, 4, and 48 hours group: n=7; 8 hours group: n=8); *P<0.05, **P<0.01, ***P<0.001 as compared with the 0‐hour group. CETP indicates cholesteryl ester transfer protein; Clec4f, C‐type lectin domain family 4; Il‐1β, interleukin‐1β; LPS, lipopolysaccharide; Ly6C, lymphocyte antigen 6 complex locus C; Mcp‐1, monocyte chemotactic protein‐1; Tnfa, tumor necrosis factor α; Vsig4, V‐set and immunoglobulin domain containing 4.

To investigate the role of hepatic macrophages in the mechanism by which LPS reduces CETP expression, we determined the regulation of CETP in relation to macrophage markers after injection of LPS in E3L.CETP mice. As expected, LPS injection caused a massive upregulation of hepatic mRNA expression of *Tnf*α 21‐fold, *Il‐1*β 19‐fold, and *Mcp‐1* (monocyte chemotactic protein‐1) 28‐fold at 4 hours after injection (all *P*<0.001, Figure 1A), indicating LPS‐induced KC activation.^6^, ^7^, ^22^ LPS also increased the expression of lipopolysaccharide‐binding protein (*Lbp*, Figure S2A). These effects were transient as gene expression returned to baseline at 48 hours after injection. LPS markedly decreased mRNA expression of *Pltp* (phospholipid transfer protein) (Figure S2B) and ATP binding cassette subfamily G member 1 *(Abcg1)* (Figure S2C) in liver, up to 48 hours after LPS injection. Simultaneously, LPS injection rapidly and markedly decreased hepatic mRNA expression of *CETP* (−75% at 8 hours after injection; *P*<0.001, Figure 1B) in E3L.CETP mice. At 48 hours after LPS injection, *CETP* expression was still reduced. In parallel, LPS markedly decreased the hepatic expression of resting KC markers *Clec4f* and *Vsig4* to a similar extent (−75% and −83% at 8 hours after injection; *P*<0.01, Figure 1B). In contrast, LPS increased hepatic mRNA expression of lymphocyte antigen 6 complex locus C (*Ly6C*), a marker of infiltrating monocytes/macrophages^23^ (+48% at 8 hours after injection; *P*<0.001, Figure 1D). Correlation analyses using data of all time points (Figure 1D) showed that hepatic *CETP* expression strongly positively correlated with the expression of *Clec4f* (*r*=0.735; *P*<0.001) and *Vsig4* (*r*=0.904; *P*<0.001), whereas hepatic expression of *CETP* inversely correlated with *Ly6C* (*r*=−0.493; *P*<0.05).

### LPS Acutely Changes Kupffer Cell Subsets But Not the Hepatic F4/80^+^ Cell Number

We next performed immunohistochemistry on liver sections and the numbers of F4/80^+^, Ly6C^+^, Clec4f^+^and CETP^+^ cells were quantified. LPS administration did not affect the hepatic total macrophage/monocyte content as evidenced by the number of F4/80^+^ cells (Figure S3A). In line with the observation that LPS markedly increased the gene expression of *Ly6C*, LPS significantly increased the number of Ly6C^+^ monocytes after 4 hours (2.6‐fold) and 8 hours (3.4‐fold) (Figure S3B). LPS decreased the number of Clec4f^+^ KC only after 48 hours (−28%; *P*<0.01, Figure S3C), which coincided with a tendency of a decreased number of CETP^+^ cells at 48 hours after injection (−20%; *P*=0.06, Figure S3D). The numbers of Clec4f^+^ cells and CETP^+^ cells were not affected at 4 and 8 hours after LPS injection, which may be explained by a relatively slow turnover of CETP protein as compared with mRNA. Collectively, these data indicate that LPS acutely decreases hepatic CETP expression accompanied by changes in KC subsets.

### LPS Reduces Plasma CETP and Transiently Increases Plasma HDL‐C Level and HDL‐C/Non‐HDL‐C Ratio

**Figure 2:**
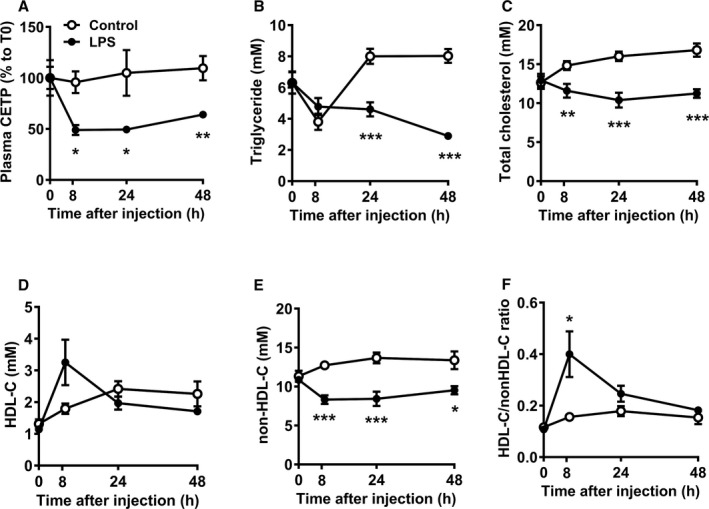
LPS reduces plasma CETP, and transiently increases plasma HDL‐cholesterol level and HDL‐C/non–HDL‐C ratio. Female APOE*3‐Leiden.CETP mice fed a Western‐type diet were intraperitoneally injected with 25 μg LPS or vehicle (control), after which blood samples were collected at the indicated time points. Plasma was assayed for CETP concentration and data are expressed relative to t=0 (A). Plasma levels of triglyceride (B), total cholesterol (C), HDL‐cholesterol (HDL‐C, D), non‐HDL‐C (E) were determined, and the ratio between HDL‐C and non–HDL‐C (F) was calculated. Data are presented as means±SEM (control and LPS group: n=7); *P<0.05, **P<0.01, ***P<0.001 as compared with the control group. CETP indicates cholesteryl ester transfer protein; HDL‐C, high‐density lipoprotein‐cholesterol; LPS, lipopolysaccharide.

Plasma CETP, lipid and lipoprotein concentrations were assayed at baseline and 8, 24, 48 hours after LPS or vehicle injection in E3L.CETP mice. LPS rapidly reduced plasma CETP concentration already at 8 hours as compared with the control group (−51%; *P*<0.01, Figure 2A), and this reduction in plasma CETP concentration persisted until 48 hours after injection, consistent with the reduced hepatic *CETP* mRNA expression. As compared with the control group, LPS significantly decreased plasma TG level after 24 and 48 hours (Figure 2B), and total cholesterol level throughout the 48 hours (Figure 2C). To investigate the effect of LPS on the distribution of cholesterol over plasma lipoproteins, HDL‐C and non–HDL‐C levels were determined. LPS transiently tended to increase HDL‐C level 8 hours after injection (+81%; Figure 2D), while persistently decreasing non–HDL‐C level throughout the 48 hours (Figure 2E). As a result, the HDL‐C/non–HDL‐C ratio transiently increased at 8 hours after LPS injection (Figure 2F), suggesting that LPS induced a rapid and transient shift of cholesterol from non–HDL lipoproteins to HDL.

### Reappearance of Hepatic CETP Expression After Elimination of Hepatic Macrophages Coincides With Reappearance of Hepatic Clec4f and Vsig4 Expression

Since LPS administration has similar time‐dependent effects on *CETP* expression and expression of the KC markers *Clec4f* and *Vsig4*, we reasoned that CETP, Clec4f and Vsig4 may be co‐expressed by a similar subset of macrophages. This would imply that, following elimination of hepatic macrophages, reappearance of hepatic CETP expression caused by monocyte infiltration would coincide with reappearance of *Clec4f* and *Vsig4*. Therefore, in a next experiment we eliminated hepatic macrophages from E3L.CETP mice by clodronate injection, without induction of inflammation,^24^, ^25^ and followed the reappearance of CETP and macrophage markers in the liver.

**Figure 3:**
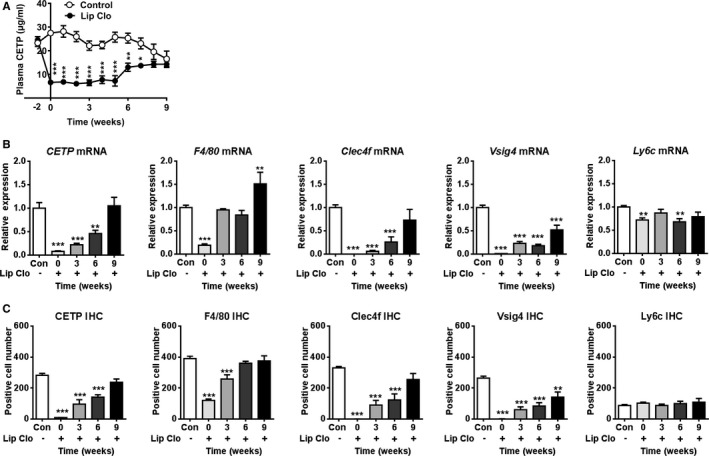
Reappearance of hepatic CETP expression after removal of hepatic macrophages coincides with reappearance of hepatic Clec4f and Vsig4 but not Ly6C. Female APOE*3‐Leiden.CETP mice fed a Western‐type diet were intraperitoneally injected with liposomal clodronate (Lip Clo) and euthanized 3 days (0), 3, 6, and 9 weeks after injection. Untreated mice were taken along as control. Blood samples were collected at the indicated time points and plasma was assayed for plasma CETP (A). Livers were assayed for mRNA (B) and protein (C) of CETP, F4/80, Clec4f, Vsig4 and Ly6C. Data are presented as means±SEM (control and 0‐week group: n=9; 3, 6, and 9 weeks group: n=7). *P<0.05, **P<0.01, ***P<0.001 compared with the control group. CETP indicates cholesteryl ester transfer protein; Clec4f, C‐type lectin domain family 4; Lip Clo, liposomal clodronate; Ly6C, lymphocyte antigen 6 complex locus C; Vsig4, V‐set and immunoglobulin domain containing 4.

**Figure 4:**
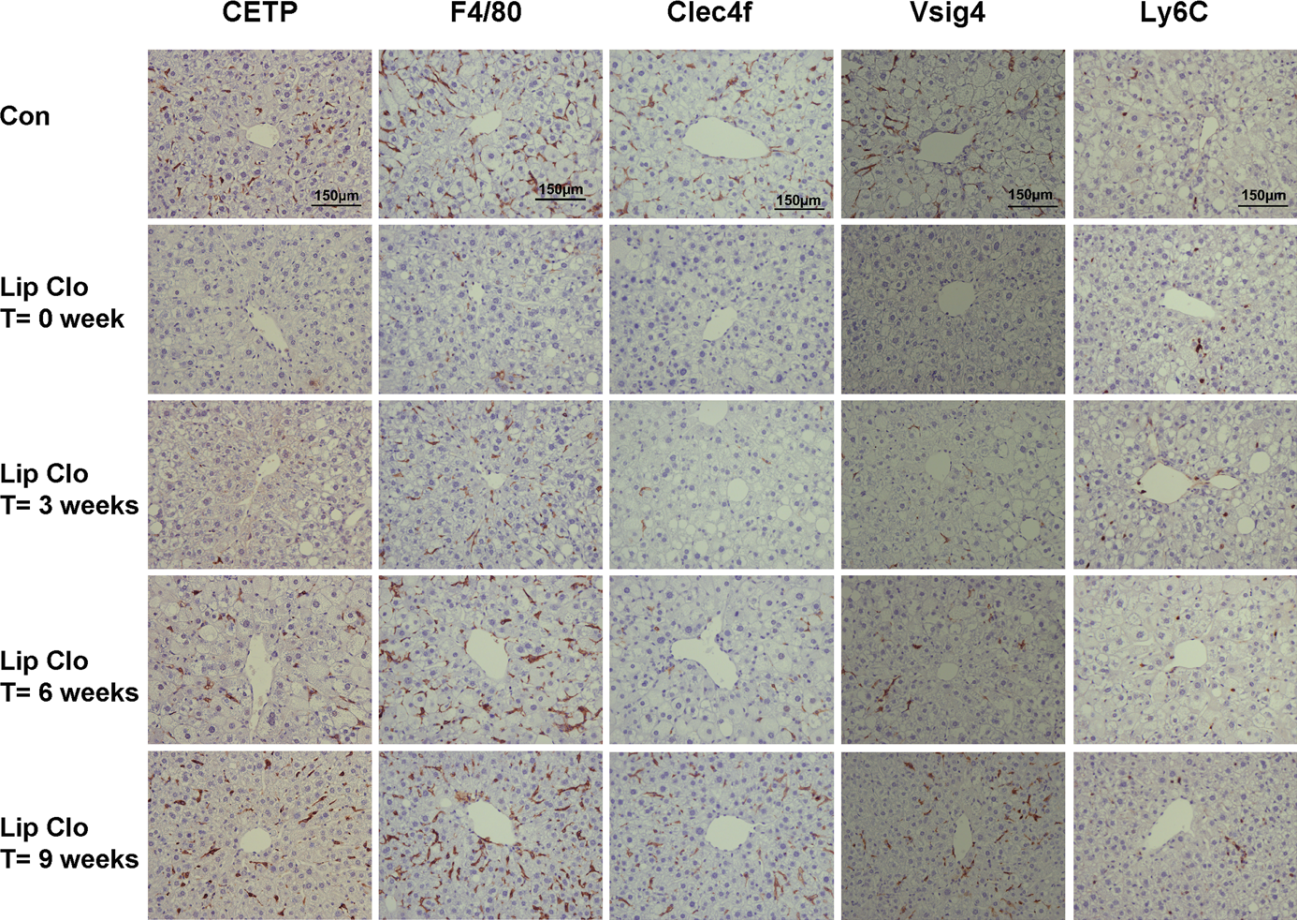
Reappearance of hepatic CETP protein and hepatic macrophage subsets after liposomal clodronate injection. Female APOE*3‐Leiden.CETP mice fed a Western‐type diet were intraperitoneally injected with liposomal clodronate (Lip Clo) and euthanized 3 days (0), 3, 6, and, 9 weeks after injection. Untreated mice were taken along as control (Con). Representative pictures of immunohistochemistry staining of CETP, F4/80, Clec4f, Vsig4 and Ly6C in liver sections from each group are shown. CETP indicates cholesteryl ester transfer protein; Clec4f, C‐type lectin domain family 4; Lip Clo, liposomal clodronate; Ly6C, lymphocyte antigen 6 complex locus C; Vsig4, V‐set and immunoglobulin domain containing 4.

In line with our previous study,^10^ liposomal clodronate rapidly and markedly decreased plasma CETP concentration compared with vehicle (−75%; Figure 3A). The plasma CETP concentration was only gradually restored to levels observed in the control group at 8 weeks after injection. Similarly, liposomal clodronate virtually depleted the liver from *CETP* mRNA (−92%), which slowly returned to baseline levels (control group) only 9 weeks after the injection. In contrast, liposomal clodronate induced an immediate decrease in *F4/80* mRNA (−82%), which was restored already 3 weeks after injection (Figure 3B). Notably, the immediate effect of liposomal clodronate treatment on mRNA expression of *Clec4f* and *Vsig4* paralleled the *CETP* mRNA expression, while liposomal clodronate had virtually no effect on *Ly6C* mRNA (Figure 3B). Furthermore, data on protein expression of the macrophage subset markers are in line with data on mRNA expression (Figures 3C and 4).

**Figure 5:**
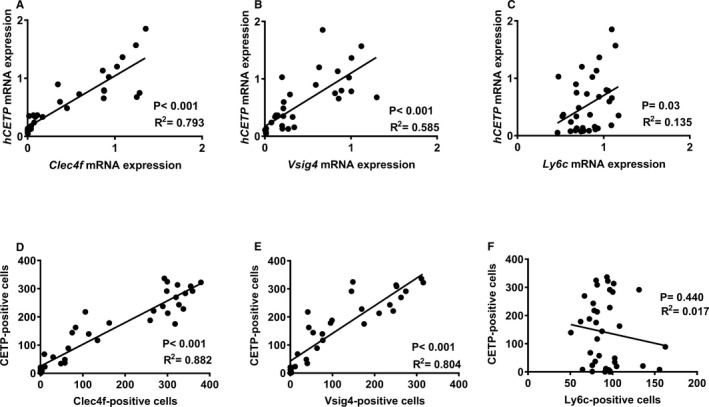
Hepatic CETP mRNA and positive cells strongly correlate with those of Clec4f and Vsig4, but not Ly6C. The correlations between hepatic CETP mRNA (A through C) or protein (D through F) with Clec4f (A and D), Vsig4 (B and E) and Ly6c (C and F) were performed, and the goodness of fit *R* ^2^ from linear regression analyses was shown. CETP indicates cholesteryl ester transfer protein; Clec4f, C‐type lectin domain family 4; Ly6C, lymphocyte antigen 6 complex locus C; Vsig4, V‐set and immunoglobulin domain containing 4.

Correlation analyses between hepatic expression of *CETP* and macrophage marker genes (Figure 5A through 5C), or the number of CETP^+^ cells and Clec4f^+^ cells, Vsig4^+^ cells, as well as Ly6C^+^ cells (Figure 5D and 5E), again showed strong positive correlations between CETP and Clec4f (Figure 5A and 5D) or Vsig4 (Figure 5B and 5E), and less clear correlation between CETP and Ly6C (Figure 5C and 5F).

### CETP is Exclusively Expressed by Clec4f^+^ Kupffer Cells

**Figure 6:**
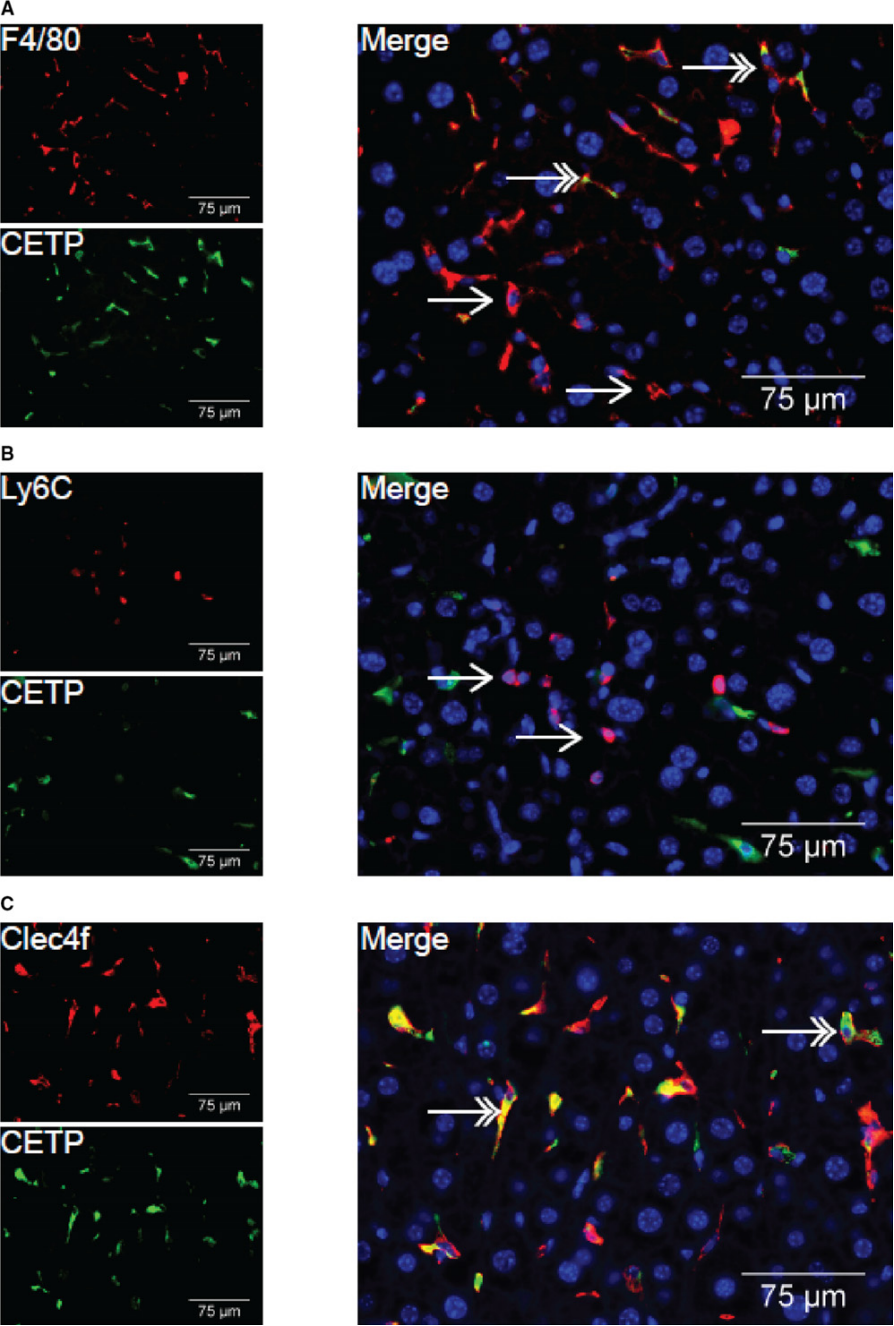
CETP protein is not co‐localized with Ly6C protein, but does co‐localize with Clec4f protein. Livers of non‐injected female APOE*3‐Leiden‐CETP mice were assayed for co‐localization of CETP and F4/80 (A), Ly6C (B) and Clec4f (C). Red; F4/80, Ly6C or Clec4f, Green; CETP, Blue; DAPI. Double headed arrows indicate co‐localization, single‐headed arrows indicate no co‐localization. CETP indicates cholesteryl ester transfer protein; Clec4f, C‐type lectin domain family 4; Ly6C, lymphocyte antigen 6 complex locus C.

To assess whether the strong correlations observed between hepatic CETP and Clec4f/Vsig4 (both mRNA expression and number of positive cells) are because of co‐expression, we next performed double immunofluorescence staining of CETP and F4/80, Clec4f, or Ly6C on liver sections. Hepatic CETP co‐localized in cells that also express F4/80, albeit that only a subset of F4/80^+^ cells (65.4±9.9%) were CETP positive (Figure 6A), which is in agreement with our previous findings.^10^ While Ly6C^+^ cells did not stain for CETP protein (only 4.4±3.9% Ly6C^+^ cells were CETP positive, Figure 6B), 94.9±8.7% Clec4f^+^ cells stained positive for CETP protein (Figure 6C).

### In Humans, Plasma CETP Concentration Correlates With Hepatic VSIG4 Expression and is Reduced by LPS Injection

**Figure 7:**
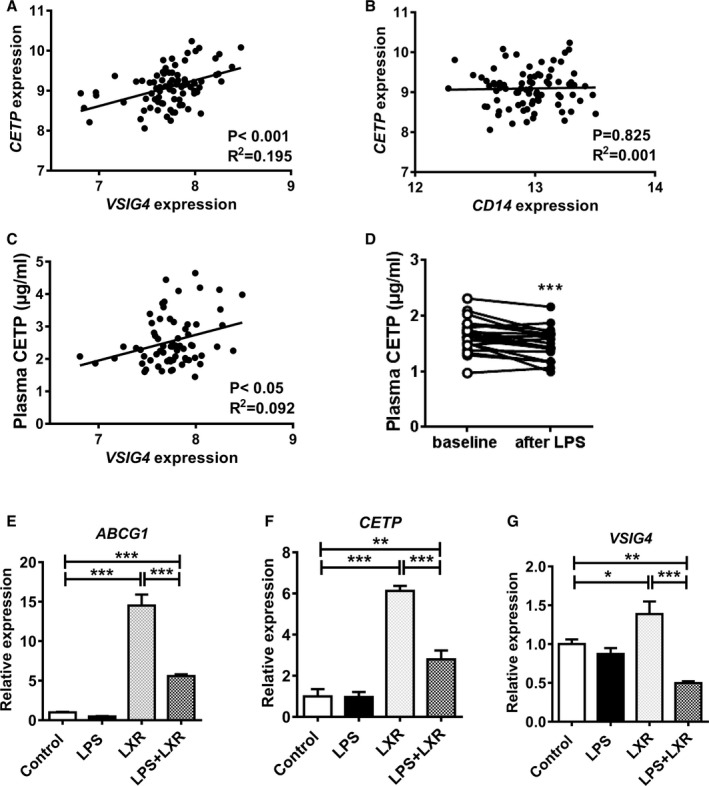
LPS reduces plasma CETP concentration that correlates with hepatic VSIG4 expression in humans. Ninety‐three liver biopsies were taken during bariatric surgery, and assayed for gene expression by microarray analysis. The correlation between the expression of *CETP* and *VSIG4* (A) and *CD14* (B), and plasma CETP concentration and *VSIG4* expression (C) were determined, and the goodness of fit *R* ^2^ from linear regression analyses was shown. D, Twenty healthy males received a bolus infusion of 1 ng/kg body weight of LPS via the antecubital vein. After an overnight fast, blood samples were collected before (baseline) and 24 hours after LPS infusion, and plasma CETP concentration was determined. ***P<0.001 as compared with the baseline. E through G, Human monocyte‐derived macrophages were treated with vehicle (control group), LPS (LPS group), TO‐901317 (LXR group) and LPS+TO‐901317 (LPS+LXR group). The mRNA expression of *ABCG1*,*CETP* and *VSIG4* was normalized to the Control group. Data are presented as means±SEM (n=4). *P<0.05, **P<0.01, ***P<0.001. ABCG1 indicates ATP binding cassette subfamily G member 1; CD14, cluster of differentiation 14; CETP, cholesteryl ester transfer protein; LPS, lipopolysaccharide; VSIG4, V‐set and immunoglobulin domain containing 4.

By using previously generated microarray gene expression data from liver biopsies of 93 subjects who underwent elective bariatric surgery,^20^ we evaluated the correlation between hepatic expressions of CETP with markers for macrophage subsets in humans. In line with findings from E3L.CETP mice, hepatic expression of *CETP* in humans correlated with *VSIG4* (*r*=0.441; *P*<0.001, Figure 7A), but not *CD14* (Figure 7B), a marker for human monocytes.^26^ Importantly, hepatic *VSIG4* expression significantly correlated with plasma CETP concentration in these subjects (*r*=0.303; *P*<0.05, Figure 7C). Adjustment for age and sex did not change these findings (not shown). Also, from a publicly available large data set of subjects undergoing bariatric surgery,^27^ we previously observed a high correlation between hepatic expression of CETP and the general macrophage marker MARCO (*r*=0.62, *P*=1.74×10^−71^).^10^ Using data from the same database, we now observed an even better correlation between the hepatic expression of *CETP* and *VSIG*4 (*r*=0.67, *P*=5.27×10^−86^) (Figure S4), which suggests that, in humans, CETP is also expressed by KCs.

To evaluate whether LPS lowers plasma CETP levels in humans similarly as in E3L.CETP mice, we determined CETP levels in 20 healthy males just before and 24 hours after administration of LPS.^21^ Indeed, we observed that LPS also decreases plasma CETP concentration in humans (−10%; *P*<0.001, Figure 7D).

### LPS Stimulation Decreases the LXR‐Mediated Upregulation of CETP and VSIG4

In search for the underlying mechanism why KCs show decreased CETP expression in response to LPS, we performed an in vitro experiment using human peripheral blood monocyte‐derived macrophages. As expected, treatment with the liver X receptor (LXR) agonist TO‐901317 strongly increased mRNA expression of the target genes *ABCG1* (15‐fold, Figure 7E) and *CETP* (6‐fold, Figure 7F). Interestingly, stimulation with LPS decreased the LXR‐mediated upregulation of *ABCG1* (Figure 7E) and *CETP* (Figure 7F), while LPS alone had no effect. Notably, the LXR agonist also increased the expression of *VSIG4* (+39%; *P*<0.05), which was similarly counteracted by LPS stimulation (Figure 7G). Collectively, these data suggest that LPS decreases the LXR‐mediated upregulation of CETP and VSIG4 in KCs.

## Discussion

In the present study, we investigated the role of KC subsets in the mechanism by which LPS reduces hepatic CETP expression and plasma CETP concentration. Our data showed that LPS rapidly and markedly reduces hepatic expression of CETP in parallel with KC activation and reduced expression of resting KC markers, without affecting the hepatic macrophage content. The reduction in hepatic CETP expression caused a decrease in plasma CETP concentration, and consequently a shift of plasma cholesterol from non‐HDL lipoproteins to HDL. We next confirmed that hepatic CETP is exclusively confined to the resting KC subset (ie, F4/80^+^Ly6C^−^Clec4f^+^Vsig4^+^), while being absent from immature monocytes and/or activated KCs in the liver (ie, F4/80^+^Ly6C^+^Clec4f^−^Vsig4^−^). Our data indicated that LPS regulates CETP expression and plasma lipoprotein composition via activating the resting KC subset.

We have previously demonstrated that the liver is the main source of plasma CETP, and that hepatic macrophages are responsible for the expression of CETP in humans and CETP transgenic (E3L.CETP) mice.^10^ Furthermore, a recent study in which hepatectomized mice were repopulated with human primary hepatocytes showed that these liver‐humanized mice did not express CETP in the liver and completely lacked CETP in serum,^28^ confirming that human hepatocytes do not express CETP. In addition, we observed that not all hepatic macrophages produce CETP, as supported by the fact that in the liver only 57% of F4/80^+^ macrophages co‐express CETP in E3L.CETP mice and 39% of CD68^+^ macrophages co‐express CETP in humans.^10^ In the current study, double immunofluorescence staining of liver sections of E3L.CETP mice demonstrated that the CETP protein is confined to F4/80^+^Ly6C^−^Clec4f^+^ hepatic macrophages, and absent from F4/80^+^Ly6C^+^Clec4f^−^ macrophages. Additionally, the kinetics of the restoration of plasma CETP concentration and hepatic macrophage subsets upon liposomal clodronate injection indicated that F4/80^+^Ly6C^−^Clec4f^+^ macrophages in the liver are the predominant cellular source of the plasma CETP pool. It should be noted that F4/80 is a general marker for monocytes and macrophages including, but not restricted to, KCs.^3^, ^29^ Clec4f, also known as Kupffer cell receptor, has been identified as an exclusive marker for the resting KC.^3^, ^30^ The reappearance of the different macrophage subsets indicated that Clec4f^+^ KCs take longer to reappear in the liver than F4/80^+^ macrophages (9 weeks versus 3–6 weeks), confirming that F4/80^+^Clec4f^+^ KCs are more mature KCs than F4/80^+^Clec4f^−^ macrophages. In contrast to mice, in humans *CLEC4F* expression is not confined to liver macrophages, while *VSIG4* is exclusively expressed by resting mature KCs.^2^, ^31^ Here, we showed in mice that *Vsig4* mRNA and protein expression paralleled the reappearance of Clec4f and CETP expression. Gene expression analysis using microarrays of 93 liver biopsies obtained from bariatric surgery revealed a strong correlation between hepatic *CETP* expression and *VSIG4* expression in humans. Also, plasma CETP concentration correlates with *VSIG4* expression in human livers. Therefore, we conclude that hepatic CETP expression in humans is also confined to resting KCs, which is thus the predominant pool of plasma CETP.

We showed that a bolus injection of LPS into E3L.CETP mice rapidly reduces hepatic *CETP* expression, without affecting total hepatic macrophage content as shown by the number of F4/80^+^ cells, indicating that LPS reduced CETP expression in hepatic macrophages per se. This finding is in concordance with previous in vitro data showing that LPS, and other inflammatory stimuli, such as TNFα and interferon γ reduced *CETP* mRNA expression in bone marrow‐derived macrophages from CETP transgenic mice and in human monocyte‐derived macrophages.^32^ In fact, LPS strongly reduced *Clec4f* expression in KCs in vitro.^33^ Also, we now showed that upon LPS administration, hepatic *CETP* expression markedly positively correlates with the *Clec4f* expression, while it inversely correlates with the expression of *Ly6C* and macrophage activation markers, ie, *Tnf*α, *Il‐1*β and *Mcp‐1*. This acute‐phase rise in *Lbp* expression is probably mediated by proinflammatory cytokines, such as interleukin‐1,^34^ which has been shown to protect against LPS induced systemic inflammation.^35^ We further observed that in healthy subjects, LPS also rapidly decreases plasma CETP concentration. Given the fact that hepatic CETP expression is confined to F4/80^+^Ly6C^−^Clec4f^+^Vsig4^+^ resting mature KCs, our data indicate that LPS rapidly activates resting KCs to become Clec4f^−^ macrophages in mice, or VSIG4^−^ macrophages in human livers, simultaneously reducing hepatic CETP expression and decreasing plasma CETP concentration. The proinflammatory signals derived from activated KCs, such as TNFα, IL‐1β, and MCP‐1, may drive a vicious cycle activating F4/80^+^Ly6C^−^VSIG4^+^ KCs to lose the expression of VSIG4. Indeed, a previous study^2^ has demonstrated that VSIG4 expression is restricted to resting macrophages and that expression was completely lost in inflamed macrophages.

The CETP gene promoter contains LXR binding elements,^36^ and LXR activation strongly increases CETP gene expression and plasma CETP levels.^37^ In addition, LXR signaling plays a crucial role in driving the specialization of macrophage subsets.^38^ In pursuit of the underlying mechanism why KCs show decreased CETP expression in response to LPS, we treated human blood monocyte‐derived macrophages with an LXR agonist, LPS or both. Our data are consistent with a mechanism in which LPS reduces the LXR‐induced expression of CETP in macrophages. Interestingly, we observed that LXR activation also increased the expression of *VSIG4*, which was counteracted by LPS stimulation. Since the promoter of *VSIG4* contains no classical LXR‐responsive element,^39^ the exact mechanism underlying this upregulation is currently unknown but may involve a distal or non‐classical LXR‐responsive element. Nevertheless, it is thus likely that downregulation of CETP and VSIG4 by LPS in the macrophage are parallel events. Notably, LPS administration to mice also largely decreased the mRNA expression of the LXR‐target gene *Abcg1* in liver. Since *Abcg1* mRNA expression levels in KCs is 70‐fold higher than in parenchymal hepatocytes,^40^ our in vivo data confirm that LPS stimulation decreases LXR activation in KCs.

CETP is a member of the LPS binding protein (LBP) family, which includes PLTP (phospholipid transfer protein), BPI (bactericidal permeability increasing protein) and LBP itself. While LPS decreased *CETP* expression, it increased *Lbp* expression. Interestingly, the reduction in *Pltp* mRNA expression, which is in line with previous findings,^41^ coincided with the reduction in *CETP* mRNA expression. CETP has a low binding affinity to LPS (Kd >25 mmol/L), as compared with LBP (Kd=0.8 nmol/L) and BPI (Kd=0.5 nmol/L).^42^ Therefore, CETP likely only plays a role in LPS binding in the acute phase of LPS exposure, when circulating LPS concentration is high. This may explain the observation that CETP expression markedly improves the mouse survival rate after injection of a lethal dose of LPS.^17^ In addition, CETP plays an important role in lipoprotein metabolism in humans. After secretion into the circulation, the CETP protein binds mainly to HDL, and promotes bidirectional transfer of CE, TG, and to lesser extent phospholipid between plasma lipoproteins. Upon LPS administration, reduced plasma CETP concentration results in increased HDL, which has well‐documented anti‐inflammatory properties.^43^, ^44^ A recent study showed that increasing HDL via CETP inhibition inhibited neointimal hyperplasia in balloon‐injured rabbits, that the benefit was attributed to the anti‐inflammatory properties of HDL.^45^ In fact, it has previously been demonstrated that low HDL‐C in healthy subjects was associated with an increased inflammatory response to an LPS challenge,^21^ further supporting the anti‐inflammatory role of endogenous HDL. It is thus tempting to speculate that CETP‐expressing species have increased flexibility to respond to invading Gram‐negative organisms that release endotoxin/LPS. The rapid conversion of KC subsets to lose CETP expression and subsequently increase HDL may be of importance in the defense against Gram‐negative bacterial infections.

It should be mentioned that the E3L.CETP mice express the human CETP mini‐gene under the control of its natural flanking regions.^36^ Although it cannot be excluded that some regulatory elements may be missing from this construct, this human CETP transgenic mice were shown to respond in a human‐like fashion to LXR agonism^37^ and FXR agonism.^46^ Notably, in E3L.CETP mice, the markedly increased HDL‐C/non–HDL‐C ratio was already normalized at 48 hours after LPS injection, whereas plasma CETP level and hepatic CETP mRNA expression were still lower at this time point. Moreover, in healthy subjects, 24 hours after LPS injection, they had decreased plasma TG^21^ and CETP concentration as shown in the present study, while plasma HDL‐C was not changed.^21^ Together, this indicates that LPS exposure not only affects CETP expression but also other pathways involved in lipoprotein metabolism.^47^

In conclusion, our findings show that hepatic CETP is exclusively expressed by resting KCs (ie, F4/80^+^Ly6C^−^Clec4f^+^) but not by activated macrophages or monocytes in the liver. In response to inflammatory stimuli, ie, LPS exposure, resting KCs become activated and lose CETP expression. As a consequence, plasma CETP concentration is also rapidly decreased and HDL‐C is raised. This sequence of events may play a role in the host defense via the anti‐inflammatory effects of HDL. The strong association between the expression of CETP and activation markers by KCs implies that modulating HDL metabolism via CETP inhibition may affect the inflammatory status of the liver.

## Sources of Funding

This study was supported by the framework of the Center for Translational Molecular Medicine (www.ctmm.nl), project PREDICCt (grant 01C‐104) to Willems van Dijk, the EU grant FP7‐HEALTH‐305707 to Groen: “A systems biology approach to RESOLVE the molecular pathology of 2 hallmarks of patients with metabolic syndrome and its co‐morbidities; hypertriglyceridemia and low HDL‐cholesterol”, the Centre for Medical Systems Biology (CMSB) and Netherlands Consortium for Systems Biology (NCSB), both within the framework of the Netherlands Genomics Initiative (NGI)/Netherlands Organisation for Scientific Research (NWO), “the Netherlands CardioVascular Research Initiative: the Dutch Heart Foundation, Dutch Federation of University Medical Centres, the Netherlands Organisation for Health Research and Development and the Royal Netherlands Academy of Sciences” for the GENIUS project “Generating the best evidence‐based pharmaceutical targets for atherosclerosis” (CVON2011‐19). PCN Rensen is an Established Investigator of the Dutch Heart Foundation (2009T038). Wang is supported by a VENI grant from NWO‐ZonMW (91617027). Fu is supported by a NWO‐VIDI grant (864.13.013) and CardioVasculair Onderzoek Nederland (CVON 2012‐03).

## Disclosures

None.

## Supporting information


**Table S1.** Primers Sequences Use for RT‐Qpcr
**Figure S1.** CETP staining in mouse and human livers. Representative picures of IHC staining of CETP protein in liver sections of (A) non‐CETP transgenic mice (APOE*3‐Leiden mice), (B) APOE*3‐Leiden.CETP transgenic mice, and (C) a healthy human donor.
**Figure S2.** LPS acutely increases hepatic *Lbp* expression and decreases *Pltp* and *Abcg1* expression. Female APOE*3‐Leiden.CETP mice fed a Western‐type diet were intraperitoneally injected with 25 μg LPS, after which mice were euthanized at the indicated time points. Livers were assayed for mRNA of (A) *Lbp*, (B) *Pltp* and (C) *Abcg1*. Data are presented as means±SEM (n=7–8); **P<0.01, ***P<0.001 as compared with the 0‐hour group.
**Figure S3.** LPS acutely changes hepatic macrophage subsets rather than macrophage number. Female APOE*3‐Leiden.CETP mice fed a Western‐type diet were intraperitoneally injected with 25 μg LPS, after which mice were euthanized at the indicated time points. Livers were assayed for F4/80‐positive macrophages (A), Ly6C‐positive monocytes (B), Clec4f‐positive Kupffer cells (C) and CETP‐positive cells (D). Data are presented as means±SEM (n=7–8); **P<0.01, ***P<0.001 as compared with the 0‐hour group.
**Figure S4.** Hepatic *CETP* expression correlates with *VSIG4* expression in humans. Scatter plots of the correlation between the expression of *CETP* and *VSIG4* in liver was determined by using a publicly available dataset consisting of 651 subjects.^1^
