# Supplementary material for: Lipopolysaccharide Lowers Cholesteryl Ester Transfer Protein by Activating F4/80+Clec4f+Vsig4+Ly6C− Kupffer Cell Subsets
Source: J Am Heart Assoc. 2018 Mar 10;7(6):e008105. doi: 10.1161/JAHA.117.008105 (PMC5907564; doi:10.1161/JAHA.117.008105)
Supplement: Supplementary file 1 — Table S1. Primers Sequences Use for RT‐Qpcr Figure S1. CETP staining in mouse and human livers. Representative picures of IHC staining of CETP protein in liver sections of (A) non‐CETP transgenic mice (APOE*3‐Leiden mice), (B) APOE*3‐Leiden.CETP transgenic mice, and (C) a healthy human donor. Figure S2. LPS acutely increases hepatic Lbp expression and decreases Pltp and Abcg1 expression. Female APOE*3‐Leiden.CETP mice fed a Western‐type diet were intraperitoneally injected with 25 μg LPS, after which mice were euthanized at the indicated time points. Livers were assayed for mRNA of (A) Lbp, (B) Pltp and (C) Abcg1. Data are presented as means±SEM (n=7–8); **P<0.01, ***P<0.001 as compared with the 0‐hour group. Figure S3. LPS acutely changes hepatic macrophage subsets rather than macrophage number. Female APOE*3‐Leiden.CETP mice fed a Western‐type diet were intraperitoneally injected with 25 μg LPS, after which mice were euthanized at the indicated time points. Livers were assayed for F4/80‐positive macrophages (A), Ly6C‐positive monocytes (B), Clec4f‐positive Kupffer cells (C) and CETP‐positive cells (D). Data are presented as means±SEM (n=7–8); **P<0.01, ***P<0.001 as compared with the 0‐hour group. Figure S4. Hepatic CETP expression correlates with VSIG4 expression in humans. Scatter plots of the correlation between the expression of CETP and VSIG4 in liver was determined by using a publicly available dataset consisting of 651 subjects.1 [file JAH3-7-e008105-s001.pdf]

SUPPLEMENTAL MATERIAL

Table S1. Primers sequences use for RT-Qpcr.

Gene	Forward primer	Reverse primer
ABCG1	CAGGAAGATTAGACACTGTGG	GAAAGGGGAATGGAGAGAAGA
Abcg1	AGGTCTCAGCCTTCTAAAGTTCCTC	TCTCTCGAAGTGAATGAAATTTATCG
βactin	AACCGTGAAAAGATGACCCAGAT	CACAGCCTGGATGGCTACGTA
β-2m	*TGACCGGCTTGTATGCTATC*	*CAGTGTGAGCCAGGATATAG*
CETP	CATGTCTCGGCTCGAGGTAG	TTCTGCTACAAGCCCCATCC
Clec4f	ACTGAAGTACCAAATGGACAATGTTAGT	GTCAGCATTCACATCCTCCAGA
F4/80	CTTTGGCTATGGGCTTCCAGTC	GCAAGGAGGACAGAGTTTATCGTG
Hprt	TTGCTCGAGATGTCATGAAGGA	AGCAGGTCAGCAAAGAACTTATAG
Il-1β	GCAACTGTTCCTGAACTCAACT	ATCTTTTGGGGTCCGTCAACT
Lbp	CCTGAGACTCGCCATCTCTGA	AGGAGGAGGTCCACTGAAATG
Ly6c	CTGCAACCTTGTCTGAGAGGA	GTCCCTGAGCTCTTTCTGCAC
Mcp-1	GCATCTGCCCTAAGGTCTTCA	TTCACTGTCACACTGGTCACTCCTA
Pltp	TCAGTCTGCGCTGGAGTCTCT	AAGGCATCACTCCGATTTGC
Tnfα	AGCCCACGTCGTAGCAAACCAC	TCGGGGCAGCCTTGTCCCTT
Vsig4	TCACCTATGGCCACCCCACC	AGGCGGCCTCTGTACTTTGCCT
VSIG4	CACTGACATGGATGGCTACCT	AAGACAGGCAGGCTCTTTCC

Figure S1. CETP staining in mouse and human livers.

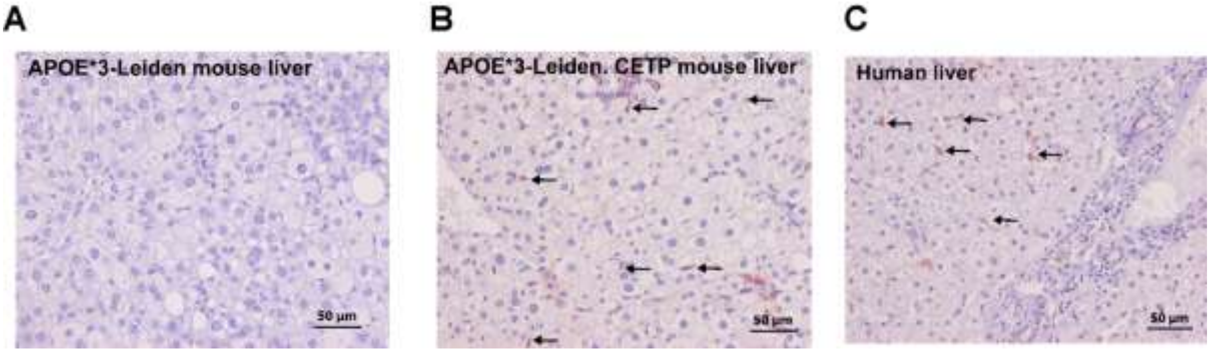

Representative picures of IHC staining of CETP protein in liver sections of (**A**) non-CETP transgenic mice (APOE*3-Leiden mice), (**B**) APOE*3-Leiden.CETP transgenic mice, and (**C**) a healthy human donor.

Figure S2. LPS acutely increases hepatic *Lbp* expression and decreases *Pltp* and *Abcg1* expression.

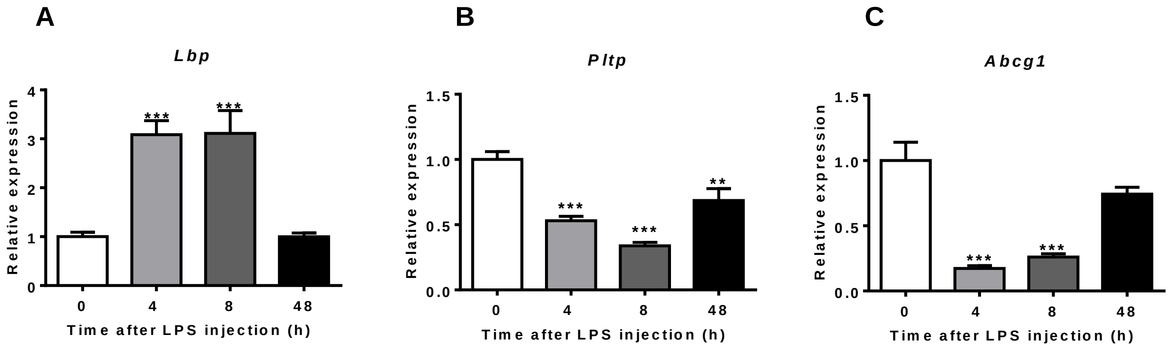

Female APOE*3-Leiden.CETP mice fed a Western-type diet were intraperitoneally injected with 25 µg LPS, after which mice were sacrificed at the indicated time points. Livers were assayed for mRNA of (**A**) *Lbp*, (**B**) *Pltp* and (**C**) *Abcg1*. Data are presented as means ± SEM (n=7-8); **P<0.01, ***P<0.001 as compared to the 0 h group.

Figure S3. LPS acutely changes hepatic macrophage subsets rather than macrophage number.

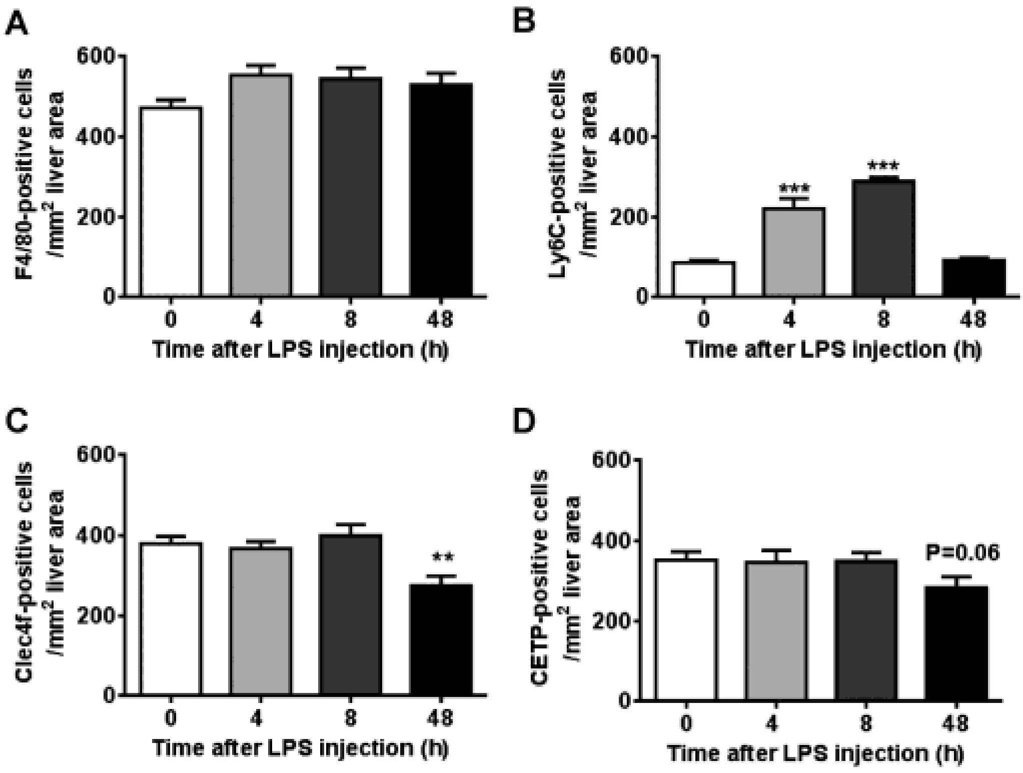

Female APOE*3-Leiden.CETP mice fed a Western-type diet were intraperitoneally injected with 25 µg LPS, after which mice were sacrificed at the indicated time points. Livers were assayed for F4/80-positive macrophages (**A**), Ly6C-positive monocytes (**B**), Clec4f-positive Kupffer cells (**C**) and CETP-positive cells (**D**). Data are presented as means ± SEM (n=7-8); **P<0.01, ***P<0.001 as compared to the 0 h group.

Figure S4. Hepatic *CETP* expression correlates with *VSIG4* expression in humans.

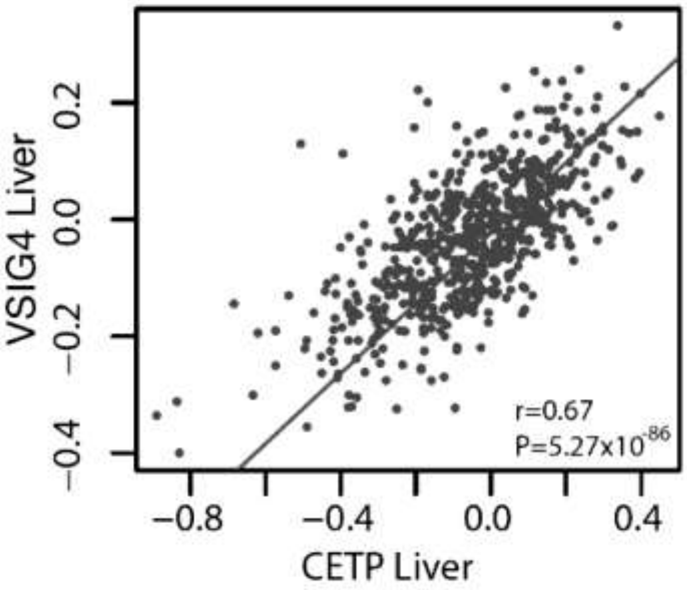

Scatter plots of the correlation between the expression of *CETP* and *VSIG4* in liver was determined by using a publicly available dataset consisting of 651 subjects[1].

Supplemental Reference:

1. Greenawalt DM, Dobrin R, Chudin E, Hatoum IJ, Suver C, Beaulaurier J, Zhang B, Castro V, Zhu J, Sieberts SK, Wang S, Molony C, Heymsfield SB, Kemp DM, Reitman ML, Lum PY, Schadt EE, Kaplan LM. A survey of the genetics of stomach, liver, and adipose gene expression from a morbidly obese cohort. *Genome Res*. 2011;21:1008-16.
